# Computational Fluid Dynamics Modeling of Liver Radioembolization: A Review

**DOI:** 10.1007/s00270-021-02956-5

**Published:** 2021-09-13

**Authors:** Jorge Aramburu, Raúl Antón, Macarena Rodríguez-Fraile, Bruno Sangro, José Ignacio Bilbao

**Affiliations:** 1 Universidad de Navarra, TECNUN Escuela de Ingeniería, 20018 Donostia-San Sebastián, Spain; 2 IdiSNA, Instituto de Investigación Sanitaria de Navarra, 31008 Pamplona, Spain; 3 Department of Nuclear Medicine, Clínica Universidad de Navarra, 31008 Pamplona, Spain; 4 Liver Unit, Clínica Universidad de Navarra and CIBEREHD, 31008 Pamplona, Spain; 5 Department of Radiology, Clínica Universidad de Navarra, 31008 Pamplona, Spain

**Keywords:** Computational fluid dynamics, Radioembolization, Therapy planning, Personalized medicine, Liver cancer, Dosimetry, Hemodynamics

## Abstract

Yttrium-90 radioembolization (RE) is a widely used transcatheter intraarterial therapy for patients with unresectable liver cancer. In the last decade, computer simulations of hepatic artery hemodynamics during RE have been performed with the aim of better understanding and improving the therapy. In this review, we introduce the concept of computational fluid dynamics (CFD) modeling with a clinical perspective and we review the CFD models used to study RE from the fluid mechanics point of view. Finally, we show what CFD simulations have taught us about the hemodynamics during RE, the current capabilities of CFD simulations of RE, and we suggest some future perspectives.

**Supplementary Information:**

The online version contains supplementary material available at 10.1007/s00270-021-02956-5.

## Background

According to GLOBOCAN database, liver cancer was the third leading cause of cancer deaths worldwide in 2018 [1]. The most common type of primary liver cancer is hepatocellular carcinoma (HCC), representing approximately 90% of all cases [2, 3]. Yttrium-90 (Y-90) radioembolization (RE) is a safe and effective treatment for patients with HCC, and it consists of the catheter-based intraarterial infusion of radioactive microspheres, which are transported by the hepatic arterial flow toward tumors, while ideally sparing healthy liver parenchyma [4]. Improvement in technical and technological aspects of RE resulted in a treatment that can be used in HCC patients in all Barcelona Clinic Liver Cancer stages [5]. Depending on the stage, the treatment intent can be curative, it can provide a bridge therapy to transplant, it can downstage to potential surgery, and it can be palliative [6–8].

A recent survey carried out among the members of the Cardiovascular and Interventional Radiological Society of Europe (CIRSE) has shown the current status of RE in Europe [9]. In addition to showing the current practice, the survey also shows the opinion of the centers that completed the survey on how RE could be improved. The four developments appearing in the questionnaire (the possibility to adapt the distribution of microspheres during the treatment based on real-time imaging feedback on dose distribution; better insight in the distribution of the microspheres in the liver; improved options to calculate the amount of activity to be injected; improved catheter designs) could be developed using computer simulation-based approaches. These and other developments could be explored using computational techniques, i.e., using computer simulations. For example, a recent study has confirmed that computer simulations of hepatic artery hemodynamics and radioactive microsphere transport can predict the segment-to-segment microsphere distribution [10].

Over the last decade, a number of studies have been published in the literature on the analysis of hemodynamics during RE via computational modeling [10–34]. The present paper reviews the studies that have studied RE from the fluid mechanics point of view. The main objectives of this review are to introduce the computational fluid dynamics (CFD) modeling tool to clinicians and show the capabilities of this tool, and to show the researchers involved in the use of CFD for improving RE various models that have been used so far. To do so, we first introduce the concept of CFD by explaining the basics of this modeling technique, so that clinicians better understand a research tool that is becoming increasingly important in this field. Then, we present various models that have been developed for the study of RE. Finally, we show what CFD simulations have taught us about the hemodynamics during RE, the current capabilities of CFD simulations of RE, and we suggest some future perspectives.

## Computational Fluid Dynamics

In short, CFD is a computer simulation tool that is based on numerical techniques and is used to predict the movement of fluids. This explanation about CFD focuses on its use to study blood flow in hepatic arteries, with the intention of being accessible to clinicians.

Blood is going to be assumed a constant-density and constant-temperature fluid, which is a valid approach when simulating hemodynamics and microsphere transport during RE. The motion of fluids, i.e., the flow, is a phenomenon that is complex, and so are the mathematical equations that describe it. This type of flows is defined by the fluid pressure and the fluid velocity vector within a spatial three-dimensional (3D) region (e.g., an artery), and a temporal framework (e.g., a cardiac cycle). Because of the nature of the equations, these must be solved using CFD. In every CFD simulation, there are three steps to be followed: the modeling, the discretization, and the solving. Each of the three steps must be carried out carefully so that the errors produced in each step are kept to a minimum and CFD simulation results can be taken as a valid representation of the real fluid flow phenomena.

**Fig. 1:**
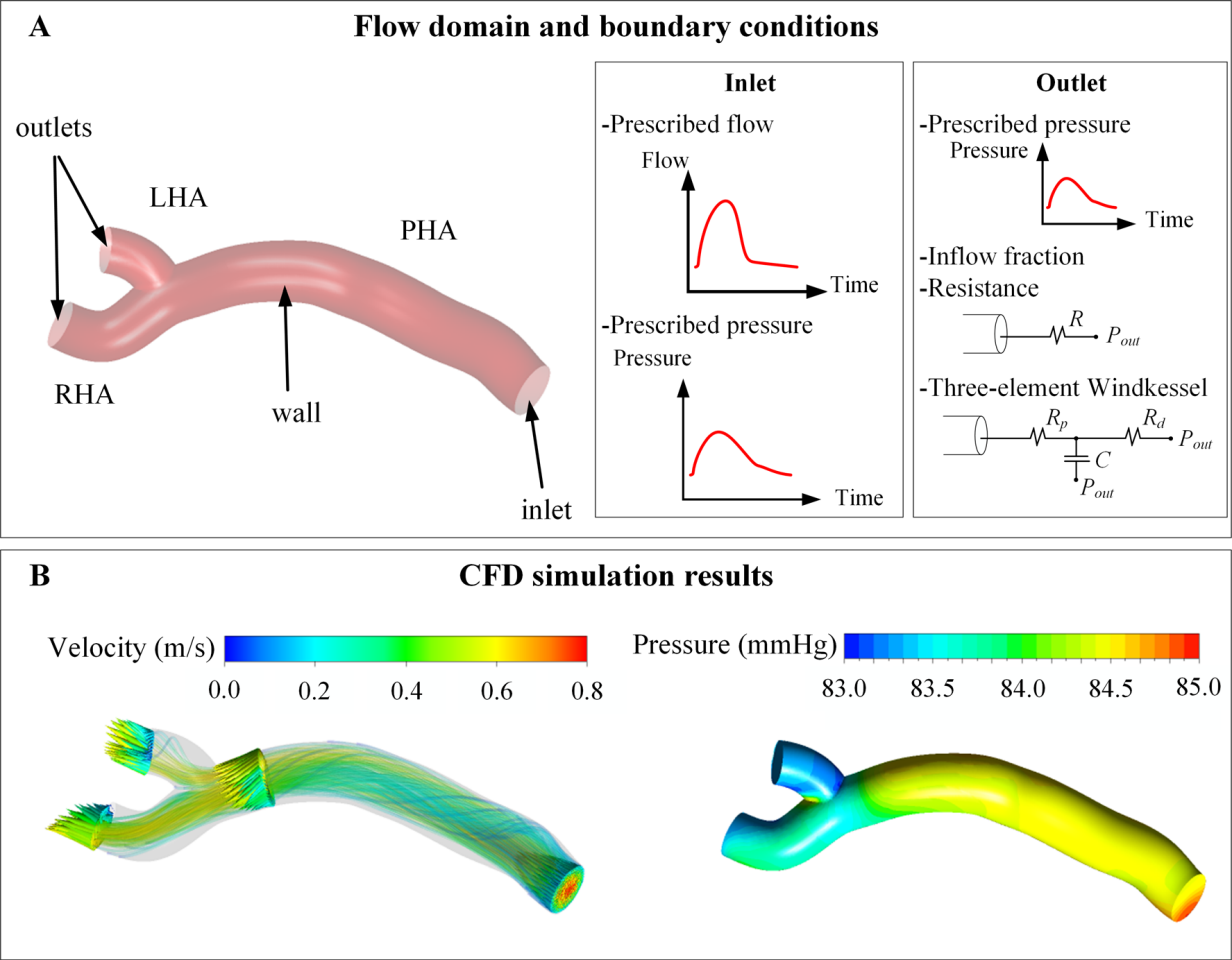
Computational fluid dynamics (CFD) simulations. **A** Flow domain and boundary conditions. An example is shown, where the flow domain is a hepatic artery bifurcation and the boundary conditions consist of an inlet, two outlets, and the artery wall. Additionally, the most common types of inlet and outlet boundary conditions are provided. At an inlet: a prescribed time-varying flow or pressure. At an outlet, a prescribed time-varying (or constant) pressure, an inflow fraction (the amount of flow exiting the outlet), and a pressure–flow relation (a resistance or a 3-element Windkessel model). **B** Example of a CFD simulation results in terms of velocity and pressure. Velocity vectors are shown in four cross-sections, together with the flow streamlines, colored with the velocity magnitude. *LHA* left hepatic artery, *PHA* proper hepatic artery, *RHA* right hepatic artery

Modeling refers to the step in which the model is built. The model consists of three components: the governing equations, the flow domain, and the boundary conditions. The governing equations are the equations that define the movement of fluids, that is, the equations of conservation of mass and conservation of linear momentum—the application of Second Law of Newton to fluid flows—and they are defined for all spatial and temporal points within the 3D region and temporal framework under study. The flow domain is the geometric space where the governing equations are going to be solved, for example, a three-branch arterial tree consisting of a proper hepatic artery (PHA), and the bifurcation of the PHA into the left hepatic artery (LHA) and right hepatic artery (RHA) (Fig. 1A). The boundary conditions are the pressure- and velocity-related characteristics of the fluid flow at the boundaries of the flow domain. In the previous example, the boundaries of the flow domain are an inlet (at the PHA level), two outlets (at the LHA and RHA levels), and the artery wall (Fig. 1A). The inlet and outlet boundary conditions represent all the vasculature that is upstream from the inlet and all the vasculature that is downstream from the outlets. Discretization refers to the step in which the complex equations of the model are converted into a series of equations that are in a suitable form to be solved in a computer. Finally, solving refers to the step in which the simulation is run and the equations are solved.

Simulation results consist of the pressure and velocity vector fields (Fig. 1B), which can be post-processed to obtain additional hemodynamic indices (e.g., the wall shear stress). It is worth noting that even if a relatively simple example is used to help better understand this explanation, CFD is currently used to solve the hemodynamics in patient-specific tortuous and intricate hepatic arteries with diameters ranging from around five millimeters to around one millimeter.

## Modeling Approaches to Study RE

CFD modeling was expected to be important in the biomedical engineering field [35], and indeed, it has become an important research tool in the field of cardiovascular modeling [36]. In the study of RE, a number of computational studies have been published in the last decade [10–34]. These studies are encapsulated in a supplementary Table S1. In this review, we focus only on the specific choices made by investigators when defining the model (governing equations, flow domain, and boundary conditions) because this choice affects the extent to which the model represents the real-life phenomenon to be analyzed. The specificity of this section intends to be helpful for the researchers immersed or to be immersed in the research of RE using CFD.

### Governing Equations

Regarding the temporal framework, both steady (time-independent) and transient (time-dependent) models have been built, by prescribing steady or pulsatile (i.e., transient) inflow and outflow boundary conditions (Table S1). This choice depends on the specific study or question to answer, but microsphere distribution depends to an extent on local near-catheter-tip hemodynamic characteristics, which are pulsatile, so in general transient CFD models should be used when calculating patient-specific microsphere distributions.

Blood has been assumed to have a density of 1060 kg/m^3^ [12, 18, 21–25, 27, 29–32] or 1050 kg/m^3^ [10, 13–17, 20, 26, 33, 34, 37]. Blood densities around 1050 kg/m^3^ have been measured in the arterial level, decreasing to a value of 1040 kg/m^3^ at the capillary level [38]. In terms of viscosity, Newtonian behavior (constant viscosity) and non-Newtonian behavior (shear rate-dependent) have been assumed. Most of the studies use a non-Newtonian fluid to determine the apparent viscosity of blood by using a modified Quemada model [22, 39]. However, some studies use a Newtonian fluid with a constant viscosity of 0.004 Pa·s [18, 21, 24, 25, 27] or 0.00309 Pa·s [40] (the asymptotic value of the modified Quemada model). At the arterial level where these simulations are carried out, blood exhibits a Newtonian behavior under normal conditions [41]. However, a more general behavior such as the modified Quemada model can be useful in cases where small arteries (diameters around 1 mm or smaller) are simulated or in cases where the blood flowrate is reduced or even completely stopped when using micro-balloon catheters, resulting in increased apparent viscosity because of red blood cell (RBC) rouleaux formation and aggregation. Even though the density and the viscosity model have been taken from the literature in all CFD-based studies, patient-specific densities and viscosities could be measured and the modified Quemada model could be calibrated. The influence of these two blood properties has not been analyzed yet, but CFD simulation results might not depend considerably on these two properties and calibrating these properties could be time-consuming.

Regarding microspheres, in the studies reported in this review, resin and glass microspheres have been modeled, even though holmium-166-poly-l-lactic acid microspheres are also commercially available and these could also be simulated by specifying their diameter and density [42]. Microsphere tracking has been usually done by applying the Second Law of Newton to microspheres. Several forces have been considered, although not all of them are always used: drag force, pressure-gradient force, gravitational force, and virtual mass force (Table S1). Another approach that has been taken regarding microspheres is that they distribute as the blood flow does [19, 21, 24, 25, 27].

### Flow Domain

Arteries have usually been considered 3D and patient-specific, patient-inspired, representative or idealized (Table S1), although reduced-order one-dimensional [34] and zero-dimensional [19, 40] geometries have also been considered. Three-dimensional models allow for detailed hemodynamic analyses in the three *x*-, *y*-, and *z*-coordinates. One-dimensional models allow for the study of pulse wave propagation study in the axial coordinate. Zero-dimensional models allow for the study of pressure and blood flowrate calculation at some specific locations.

The geometry of the catheter has not been included in the flow domain in many studies, even though microspheres are injected with a catheter (Table S1). In those cases, particle release maps were usually analyzed. A particle release map is a computer-generated colored injection cross-sectional area, with each color indicating the outlet targeted by microspheres injected at each spatial point. Both including the catheter and not including it can be valid approaches, depending on the question to be answered. For example, it is necessary to include the catheter in patient-specific simulations or to analyze the influence of the catheter on the hemodynamics near the catheter tip, but it is not essential to include the catheter to study the influence of the injection location within a cross-sectional area of a hepatic artery on the microsphere distribution.

### Boundary Conditions

At the inlets of the flow domains, pulsatile or steady velocity or pressure profiles have been prescribed (Table S1). Physiologically realistic pulsatile waveforms and even patient-specific waveforms have been used in a few cases (Fig. 1A).

At the outlets of the domain, velocity-based or pressure-based boundary conditions have been prescribed. Velocity-based boundary conditions consist of the prescription of inflow fractions (i.e., a percentage of the total inflow). This can be done using Murray’s law (distribution according to outlet branches’ diameters) or using a perfusion-based blood flow distribution [13]. Pressure-based boundary conditions can be enforced by using prescribed pressure waveforms, or by using pressure–flow relations such as a hydraulic resistance or a three-element Windkessel model (Table S1, Fig. 1A) [43]. A three-element Windkessel model models the downstream vasculature with two resistances, a compliance, and a pressure (*R*_*p*_ and *R*_*d*_, *C*, and *P*_out_ in Fig. 1A).

Artery walls have been assumed as impermeable walls with the no-slip condition, meaning that the relative velocity of the artery wall and the blood in contact with the wall is zero. Although hepatic artery walls are not strictly rigid, the rigid assumption has been taken against the compliant assumption (i.e., purely resistive non-compliant vessels) because of reduced simulation times and minor differences in simulation results [32].

## Discussion on Clinical Implications

### Can we Trust CFD Simulation Results?

In order to use CFD simulations as a reliable research tool, these simulation results must be validated, meaning that the results must be compared with reality (i.e., in vivo), or at least with an experiment (i.e., in vitro). In this regard, both in vitro validations [23, 44] and an in vivo validation have been performed [10]. The model in vivo validated consists of injecting microspheres during one cardiac cycle and simulating additional cardiac cycles so that most of the injected microspheres exit the flow domain. The predicted segment-to-segment microsphere distribution for the actual treatment is extrapolated from this one-cardiac-cycle injection. Blood flow redistribution due to microembolic effect [45] of microspheres and possible stasis scenarios are not considered, which should be assessed with changes in the boundary conditions of the CFD model. In that in vivo validation study, three patients and a total of six Y-90 resin microsphere infusions were analyzed [10]. The segment-to-segment microsphere distributions predicted by CFD simulations were compared with the segment-to-segment Y-90 PET/CT-based activity distributions measured post-RE. In order to calibrate each CFD model, first the patient-specific geometry was reconstructed to define the flow domain. To define the arterial perfusion-based boundary conditions [13], the results of a pre-RE perfusion CT were used. In addition, the catheter type, catheter tip location, and the injection velocity used in the CFD model were those recorded during the actual treatments. The authors conclude that the model is able to predict the segment-to-segment microsphere distribution [10]. The next steps consist of using CFD simulations as an actual pre-RE tool for optimizing the treatment by deciding the catheter to use, the catheter location, the activity to infuse, etc. and assessing its usefulness in predicting the microsphere distribution in the liver.

### What have we Learnt from CFD Studies?

Computer simulation-based research has taught us various lessons. First, the blood flow and microsphere transport in tortuous and intricate arterial trees is complex [13, 15, 25]. This can be seen even in the streamlines of the simple example illustrated in Fig. 1B. The complexity of this phenomenon leads us to the following three lessons, which can result in practical recommendations for clinicians.

**Fig. 2:**
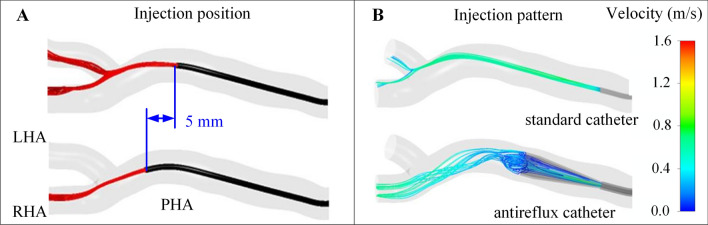
Illustration of some lessons taught by CFD-based studies. **A** Importance of catheter tip position on microsphere distribution (adapted from reference [16]). In a treatment that targets both the LHA and the RHA with the catheter placed near a bifurcation, a shift in the catheter tip as small as 5 mm results in a treatment that targets only the RHA. **B** Importance of catheter type on microsphere distribution (adapted from reference [14]). The expandable tip of the antireflux catheter makes the injection flow decelerate and the resulting injection pattern differs from that of the standard catheter. *LHA* left hepatic artery, *PHA* proper hepatic artery, *RHA* right hepatic artery

Second, the injection position (especially if near a bifurcation) is of utmost importance in predicting the microsphere distribution (Fig. 2A) [16, 22, 23]. Injection velocity can also be important if that is much greater than that of the surrounding blood flow [29]. From the hemodynamics point of view and based on the results of CFD studies, the unpredictability of the microsphere distribution tends to increase as the catheter tip is placed closer to a bifurcation. Therefore, if all the downstream branches are to be targeted, we recommend injecting the microspheres at an adequate velocity (adapted to that of blood flow) and placing the catheter tip as far from a downstream bifurcation as possible. This tip-to-bifurcation distance will allow microspheres to align with the blood flow, and microspheres will tend to distribute as the blood flow does. This is particularly interesting in the case of a patient with hypervascular tumors, because the arterial blood flow toward tumors is much greater than the flow toward the liver parenchyma. Achieving a microsphere distribution that matches the blood flow split would ensure that microspheres are preferentially targeting of tumors. If a specific branch in a bifurcation is to be targeted and the catheter cannot be advanced beyond a bifurcation, we recommend injecting close to the bifurcation at a high velocity (greater than that of blood flow) with the catheter pointing at the target branch. The applicability of these recommendations will depend on the accessibility of the hepatic artery, its morphology, the type of tumors, and the distribution of tumors in the liver.

Third, the choice of catheter type (standard/antireflux/microballoon catheter) is going to influence the microsphere distribution because it affects the hemodynamics in the vicinities of the catheter tip (Fig. 2B) [12, 14]. In general, as the cross-sectional area of a flow passage increases, the flow decelerates and the velocity of the flow decreases. With an antireflux catheter, the presence of an expandable tip makes the blood flow accelerate as a result of a decrease in the cross-sectional area of the artery lumen, and the injected flow decelerates as a result of an increase in the injection cross-sectional area. This influences the microsphere incorporation into the bloodstream, where a blood–injection mixing is promoted. As a result, microspheres tend to distribute as the blood flow does. With a standard catheter, the microsphere distribution in the first bifurcation can be unpredictable, especially if the catheter tip is located near a bifurcation. Possible microsphere distributions to daughter branches range from a microsphere distribution matching blood flow split to a microsphere distribution where all microspheres target a single daughter branch. However, even if an injection results in all microspheres being directed to one of the daughter branches of the first bifurcation, microspheres can also align with the downstream flow as microspheres cross that daughter branch and before encountering the next bifurcation. Microsphere distribution would be similar to the flow split, with respect to the flow flowing through the first bifurcation’s targeted daughter branch. With a microballoon catheter, when the blood flow is completely stopped as a result of the inflation of the microballoon, a blood flow redistribution is promoted and the hemodynamics changes with respect to the normal hepatic arterial flow [37]. From the hemodynamics point of view and based on the results of CFD studies, we recommend using an antireflux catheter when the injection is to be done near a bifurcation and both daughter branches must be targeted; otherwise, the use of a standard catheter can jeopardize the targeting of both daughter branches. Moreover, the microsphere distribution will tend to match the blood flow split. Again, this is very interesting in the case of a patient with hypervascular tumors because of their increased arterial blood supply. The applicability of this recommendation will depend on considerations different from the purely hemodynamic considerations.

Fourth, with the current modeling approach, the outlet-to-outlet or segment-to-segment microsphere distribution can be predicted [17]. Regardless of the degree of vascularization of tumors, the microsphere distribution can be predicted by analyzing via CFD proximal or distal injections (e.g., at the PHA level or at a segmental artery level) in patient-specific hepatic artery trees where the smallest arteries modeled have a diameter of around one millimeter. The information regarding the amount of microspheres depositing in each liver segment can be relevant for the multidisciplinary team that plans the treatment.

Regarding the use of CFD to study RE and taking the previous four lessons into account, we suggest that the flow domain in a CFD model should consist of the patient-specific 3D hepatic arterial tree with the specific catheter included in the artery lumen. As for the boundary conditions, we recommend prescribing patient-specific measured pulsatile flow waveforms at inlets, together with patient-specific measured pulsatile pressure waveforms at outlets. These measurements can be unavailable for a patient because they were not measured during the pretreatment assessment or because they cannot be taken accurately. In that case, we recommend prescribing physiologically realistic pulsatile flow waveforms at inlets with physiologically realistic flow fractions at outlets [13], so that realistic hemodynamic characteristics are replicated.

It is important to note that this modeling approach could be simplified considerably if the injection conditions produced a microsphere distribution that matches the blood flow distribution. In that case, the CFD simulation becomes unnecessary, because the microsphere distribution can be considered similar to the flow split defined when deriving the perfusion-based boundary conditions [13], e.g., using perfusion CT [10]. The two key questions to be answered by clinicians and engineers, respectively, are: Are we interested in distributing the microspheres as the blood flow does in my specific patient? How can we produce such hemodynamic conditions?

## Future Perspectives

In this active area of ongoing research, there are currently several challenges being addressed and to be addressed before incorporating CFD models as part of the RE treatment planning in the clinical setting and in the developments proposed in the CIRSE survey [9].

- *The simulation strategy itself*: A one-cardiac-cycle injection is simulated as a surrogate of the whole treatment. This approach assumes that the hemodynamic characteristics do not change over the course of the treatment, and it has been proved to be a valid approach [10]. Nonetheless, modeling additional events such as microembolic effects, possible stasis, and other phenomena influencing the blood flow via the inflow and outflow boundary conditions could be of interest to develop a more general CFD model of RE.
- *Simulation times*: These depend on the workstation used to run the simulations, but are usually greater than 10 h only for the solving step (Table S1). A recent computational study reduced the simulation time by 60% (in one case from 48 to 9 h) by reducing the size of the flow domain with minor changes in the calculated segment-to-segment microsphere distribution [26]. Investigating additional strategies to further reduce CFD simulation times could result in CFD simulations being a valuable tool to be used during the treatment planning.
- *Intrasegmental microsphere dynamics to predict dosimetry*: Current CFD simulations of 3D blood flow allow for the prediction of the segment-to-segment microsphere distribution, which is a valuable information for the multidisciplinary team that plans the treatment. However, knowing the specific location of microspheres within the segment would be of interest, because of the impact of the number of microspheres and their deposition within the liver parenchyma on tissue radiation tolerance [46]. Once microspheres reach the segment, the microsphere–hemodynamics is governed by other phenomena different from the ones modeled in current CFD models. In the intrasegmental level, the order of magnitude of the diameter of the arteries starts to be similar to that of the diameter of RBCs and Y-90 microspheres. As a result, (i) the hemodynamics will change because of the Fahraeus–Lindqvist effect (the thickness of the plasma layer between the RBC-containing core and the arterial wall increases, which results in a decrease in the apparent viscosity) [47], (ii) the interaction between Y-90 microspheres, which is neglected in the current models, could be important, and (iii) the friction between Y-90 microspheres and the arterial wall could be important. Nonetheless, current CFD models could be coupled with models of the intrasegmental blood flow and microsphere dynamics [48]. The results of these simulations could be used for a patient-specific dosimetry planning. For example, a protocol called CFDose has been recently developed to estimate dosimetry using CFD simulation results and Y-90 physics modeling [21]. As the authors of CFDose suggest, further complete modeling of microsphere–hemodynamics from the microsphere injection location to final deposition location could improve the dosimetry prediction for patient-specific treatment planning.
- An integrated tool for CFD-based studies of RE. Currently, there is not an integrated software package to carry out the whole process of preprocessing (modeling and discretization steps), running (solving step), and postprocessing a CFD simulation and to provide clinicians with practical recommendations about the optimal treatment parameters such as the injection location, injection velocity, injection device, activity to inject, etc. Researchers should continue working on developing such a tool, because integrating all the steps in a single simulation tool or virtual environment could be of interest to incorporate CFD-based information into the treatment planning stage. Some steps have already been given. A Computational Medical Management Program has been proposed for optimal microsphere delivery [49]. This program is based on the use of a smart microcatheter and a microsphere supply apparatus that are able to control the catheter-tip location on the injection cross-sectional plane [50] and control the injection pattern and time in the cardiac cycle [30], respectively.

This computer simulation-based research strategy has also been used to study other liver-directed transcatheter intraarterial therapies such as balloon-occluded transarterial chemoembolization [37, 40], and it could potentially be used for other transcatheter endovascular therapies.

In conclusion, in this review we have explained the basics of CFD modeling with a clinical perspective, we have encapsulated the computational studies on RE that are available in the literature, and we have shown what we have learnt from those studies. Additionally, the current capabilities and some future perspectives of CFD in the study of RE and other transcatheter endovascular therapies are indicated.

## Supplementary Information

Below is the link to the electronic supplementary material.

Supplementary file1 (PDF 569 KB)

## Abbreviations

3D: Three-dimensional
CIRSE: Cardiovascular and Interventional Radiological Society of Europe
CFD: Computational fluid dynamics
HCC: Hepatocellular carcinoma
LHA: Left hepatic artery
PHA: Proper hepatic artery
RBC: Red blood cell
RE: Radioembolization
RHA: Right hepatic artery
Y-90: Yttrium-90
