# Supplementary material for: Computational Fluid Dynamics Modeling of Liver Radioembolization: A Review
Source: Cardiovasc Intervent Radiol. 2021 Sep 13;45(1):12–20. doi: 10.1007/s00270-021-02956-5 (PMC8716346; doi:10.1007/s00270-021-02956-5)
Supplement: Supplementary file 1 — Supplementary file1 (PDF 569 KB) [file 270_2021_2956_MOESM1_ESM.pdf]

Table S1 Computational studies on liver radioembolization. Other computational studies on the hepatic arterial hemodynamics are also included.

**Study**	**Geometry type (dimensions, outlets)**	**Catheter (Yes/No)**	**Blood model**	**Artery walls**	**Blood–microsphere interaction**	**Microsphere dynamics**	**Inflow boundary conditions**	**Outflow boundary conditions**	**Software package used**	**Simulation time per simulation***
Kennedy et al. [11].	Repr. (3D, 5).	No.	Non-Newtonian [39].	Rigid.	One way.	DF.	Steady and parabolic velocity.	Steady and uniform pressures.	CFX (Ansys Inc.).	NA.
	Comments: *Microsphere release distribution (parabolic vs. uniform) and position greatly impacts the outlet-to-outlet microsphere distribution.*									
Basciano et al. [22].	Repr. (3D, 5).	No.	Non-Newtonian [39].	Rigid.	One way.	PF, GF, DF.	Pulsatile and parabolic velocity.	Pulsatile and uniform pressure profiles, and inflow fraction on one of the outlets.	CFX (Ansys Inc.).	72.87 h.
	Comments: *Temporal and cross-sectional spatial locations impact the outlet-to-outlet microsphere distribution.*									
Basciano et al. [28].	Repr. (3D, 5) and pat.-ins. (3D, 3).	Yes and no.	Non-Newtonian [39].	Rigid.	One way.	PF, GF, DF.	Pulsatile velocities and WK models.	Pulsatile pressures and WK models.	CFX (Ansys Inc.)	Days to weeks
	Comments: *This review of the previous work by the authors* [11,22] *concludes that a new catheter could be designed for optimal microsphere delivery for tumor targeting.*									
Kleinstreuer et al. [29].	Repr. (3D, 5).	Yes.	Non-Newtonian [39].	Rigid.	One way.	PF, GF, DF.	Pulsatile velocity.	Pulsatile pressure and inflow fraction on one of the outlets.	CFX (Ansys Inc.).	28.2 h-45.4 h (per pulse); 50 h-52.5 h (fluid–particle dynamics).
	Comments: *A novel smart microcatheter can optimize tumor-targeting controlling the catheter tip position, injection velocity and temporal window in the cardiac cycle, and injection function via a novel microsphere supply apparatus.*									
Childress et al. [30].	Repr. (3D, 5) and pat.-ins. (3D, 3).	Yes.	Non-Newtonian [39].	Rigid.	One way.	PF, GF, DF.	Pulsatile, parabolic velocity.	Pulsatile pressure and inflow fraction on some of the outlets.	CFX (Ansys Inc.).	NA.
	Comments: *Composite particle release maps can be used to optimize microsphere delivery to tumors using a novel smart microcatheter and a novel microsphere supply apparatus.*									

Childress et al. [31].	Repr. (3D, 5).	No.	Non-Newtonian [39].	Rigid.	One way.	PF, GF, DF.	• Pulsatile velocity.<br>• 27 steady velocity.<br>• Steady velocity.	• Pulsatile pressures.<br>• 27 steady pressures.<br>• Steady pressures.	CFX (Ansys Inc.).	• Transient: 245.46 h;<br>• Multiple steady: 18.21 h<br>• Time-averaged steady: 0.63 h
	Comments: *Simulation time can be reduced by optimizing the injection characteristics using a steady-state simulation instead of a full transient simulation.*									
Childress et al. [32].	Pat.-spec. (3D, 5).	No.	Non-Newtonian [39].	• Compliant.<br>• Rigid.	One way.	PF, GF, DF.	Pulsatile velocity.	• WK models.<br>• Pulsatile pressures.	• MFX (Ansys Inc.)<br>• CFX (Ansys Inc.)	• FSI: 3.5 days.<br>• CFD: 11-14 h.
	Comments: *CFD simulations are preferred over FSI simulations because the results are not too different and the computational time is up to 7 times smaller.*									
Aramburu et al. [33].	Pat.-spec. (3D, 29).	–	Non-Newtonian [39].	Rigid.	–	–	Pulsatile velocity.	Pulsatile pressures.	Fluent (ANSYS Inc.).	135 h.
	Comments: *A methodology to derive outflow pressure-based boundary conditions is presented, but these cannot be used in livers with tumors.*									
Umbarkar et al. [34].	Rep. (1D, 5).	No.	Non-Newtonian [39].	Compliant.	One way.	PF, DF.	Pulsatile velocity.	WK models.	Fortran.	56 min.
	Comments: *Microsphere tracking (a three-dimensional phenomenon) is done using a 1D modeling approach.*									
Xu et al. [12].	Pat.-spec. (3D, 17).	Yes (porous membrane).	Non-Newtonian [39].	Rigid.	NA.	PF, GF, DF.	Pulsatile velocity	Pulsatile pressures.	CFX (Ansys Inc.).	NA.
	Comments: *This study analyzes numerically the use of an antireflux catheter and provides a real-time infusion-stop point based on measured blood pressure.*									
Aramburu et al. [13].	Pat.-spec. (3D, 29).	–	Non-Newtonian [39].	Rigid.	–	–	Pulsatile velocity	Perfusion-based inflow fractions.	Fluent (ANSYS Inc.).	106 h and 173 h.
	Comments: *A methodology is presented to define inflow and outflow boundary conditions in hepatic artery hemodynamics simulations under cancer scenarios. The inlet's flowrate and the outlets' inflow-fractions are based on the arterial perfusion and volume values of normal- and tumor-tissues and the hepatic artery morphology.*									

Aramburu et al. [14].	Pat.-spec. (3D, 29).	Yes.	Non-Newtonian [39].	Rigid.	Two way.	PF, GF, DF, VF.	Pulsatile velocity.	Perfusion-based inflow fractions [13].	Fluent (ANSYS Inc.).	NA.
	Comments: *Injections from a sufficiently long and tortuous artery may result in a microsphere distribution that matches the blood flow distribution.*									
Aramburu et al. [15].	Pat.-spec. (3D, 29).	Yes.	Non-Newtonian [39].	Rigid.	Two way.	PF, GF, DF, VF.	Pulsatile velocity.	Perfusion-based inflow fractions [13].	Fluent (ANSYS Inc.).	NA.
	Comments: *Unintentional modifications the catheter tip location, injection velocity and catheter distal direction can result in a microsphere distribution different from the one predicted during the pretreatment workup.*									
Aramburu et al. [16].	Pat.-spec. (3D, 29).	Yes.	Non-Newtonian [39].	Rigid.	Two way.	PF, GF, DF, VF.	Pulsatile velocity.	Perfusion-based inflow fractions [13].	Fluent (ANSYS Inc.).	264.5 h.
	Comments: *Microsphere distribution can vary considerably if the catheter tip location is 5-mm-shifted between the pretreatment and the actual treatment, and this effect is even greater if the catheter tip is near a bifurcation.*									
Aramburu et al. [17].	Pat.-spec. (3D, 29).	Yes.	Non-Newtonian [39].	Rigid.	Two way.	PF, GF, DF, VF.	Pulsatile velocity.	Perfusion-based inflow fractions [13].	Fluent (ANSYS Inc.).	NA.
	Comments: *The orientation of an angled-tip catheter and the injection velocity affect microsphere distribution because the near-tip hemodynamics, which determines the microsphere travel, is altered.*									
Simoncini et al. [18].	Pat.-spec. (3D, 24), pat.-spec. (3D, 20), pat.-spec. (3D, 29), pat.-spec. (3D, 50).	–	Newtonian.	Rigid.	–	–	Steady velocity.	Diameter-based inflow fractions.	Fluent (ANSYS Inc.).	15-45 min.
	Comments: *A methodology for hepatic artery segmentation and CFD modeling is presented for application to the study of radioembolization.*									
Simoncini et al. [19].	Pat.-spec. (0D, NA).	No.	Newtonian.	Rigid.	–	(microsphere distribution = blood flow distribution.)	Steady pressure.	Steady pressure.	NA.	NA.
	Comments: *A methodology for microsphere distribution prediction during radioembolization is presented. Arteries and tumors are segmented and the hepatic artery is completed with a vascular growth law. Blood flow is modeled with a 0D model and microspheres are assumed to be distributing as the blood flow does.*									

Aramburu et al. [40].	Ideal. (0D, 8).	No.	Newtonian.	Rigid.	–	–	Steady pressure.	Resistances.	Matlab (Mathworks).	5 s.
	Comments: *Zero-dimensional modeling has been used to predict the blood flow redistribution in hepatic arteries during B-TACE. This could potentially help use an optimal balloon-occlusion site during the treatment.*									
Aramburu et al. [37].	Pat.-spec. (3D, 11).	No.	Non-Newtonian [39].	Rigid.	–	–	Steady velocity.	Perfusion-based inflow fractions [13].	Fluent (ANSYS Inc.).	NA.
	Comments: *A CFD model for B-TACE is presented, which can be used to predict the blood flow redistribution after balloon-occlusion of an artery.*									
Ortega et al. [20].	Ideal. (3D, 8).	Yes.	Non-Newtonian [39].	Rigid.	Two way.	PF, GF, DF, VF.	Pulsatile velocity.	Perfusion-based inflow fractions [13].	Fluent (ANSYS Inc.).	37.5 h.
	Comments: *In order to use an idealized hepatic artery geometry, the helicity resulting from tortuous hepatic arteries must be prescribed in the inflow boundary conditions.*									
Roncali et al. [21].	Pat.-spec. (3D, 23) and pat.-spec. (3D, 46).	No.	Newtonian.	Rigid.	One way.	(Following injection streamlines.)	Pulsatile velocity.	WK models.	SimVascular.	NA.
	Comments: *A simulation-based tool called CFDose is presented for personalized dosimetry. CFDose combines CFD modeling and Y-90 physics modeling.*									
Bomberna et al. [23].	Pat.-spec. (3D, 16) and pat.-spec. (3D, 21).	No.	Non-Newtonian [39].	Rigid.	One way.	PF, GF, DF.	Steady velocity.	Perfusion-based inflow fractions [13] and Murray's Law-based inflow fractions.	Fluent (ANSYS Inc.).	NA.
	Comments: *Clinical parameter (injection location, microsphere size, microsphere density, etc.) optimization, catheter advancement closer to the tumor-feeding arteries and catheter tip position control may enable improvement in tumor-targeting.*									
Taebi et al. [24].	Pat.-spec. (3D, 46).	No.	Newtonian.	Rigid.	One way.	(Following injection streamlines.)	Pulsatile velocity.	WK models.	SimVascular.	NA.
	Comments: *The impact of outlet boundary conditions (WK models) is assessed, concluding that this sensitivity analysis shows that a careful selection of boundary conditions has to be made because of its importance in the blood flow distribution and therefore microsphere distribution.*									

Taebi et al. [25].	Pat.-spec. (3D, 46).	No.	Newtonian.	Rigid.	One way.	(Following injection streamlines.)	Pulsatile velocity.	WK models.	SimVascular.	NA.
	Comments: *The use of the previously presented CFDose is explored, and the importance of performing patient-specific CFD simulations to better predict dosimetry is stressed.*									
Antón et al. [10].	Pat.-spec. (3D, 11), pat.-spec. (3D, 8), pat.-spec. (3D, 7).	Yes.	Non-Newtonian [39].	Rigid.	Two way.	PF, GF, DF, VF.	Pulsatile velocity.	Perfusion-based inflow fractions [13].	Fluent (ANSYS Inc.).	12 h.
	Comments: *A CFD model is in-vivo validated by comparing the computer-simulated prediction of segment-to-segment microsphere distribution with measured segment-to-segment activity distribution.*									
Lertxundi et al. [26].	Pat.-spec. (3D, 14; 6), pat.-spec. (3D, 29; 4), pat.-spec. (3D, 43; 5).	Yes.	Non-Newtonian [39].	Rigid.	Two way.	PF, GF, DF, VF.	Pulsatile velocity.	Perfusion-based inflow fractions [13].	Fluent (ANSYS Inc.).	9 h–48 h.
	Comments: *Simulation time can be reduced by reducing the size of the flow domain (geometry). In this study, an average simulation time reduction of 60% was achieved in three cases (from 23.25 h to 16.5 h, from 48 h to 9 h, and from 25.5 h to 11 h).*									
Taebi et al. [27]	Pat.-spec. (3D, NA).	Yes.	Newtonian.	Rigid.	One way.	(Following injection streamlines.)	Pulsatile velocity.	WK models.	SimVascular.	NA.
	Comments: *In the current version of SimVascular, the velocity profiles that can be prescribed are parabolic, plug, and Womersley profiles. In this study, a pipeline to include the influence of the catheter at the inlet velocity profile is provided. This velocity profile consists of the catheter flow profile (zero, constant, or parabolic) and the blood flow profile, with zero velocity at the catheter and arterial walls.*									

0D: zero-dimensional; 1D: one-dimensional; 3D: three-dimensional; B-TACE: balloon-occluded transarterial chemoembolization; CFD: computational fluid dynamics; DF: drag force; FSI: fluid–structure interaction; GF: gravitational force; Ideal.: idealized; NA: not available; Pat.-ins.: patient-inspired; Pat.-spec.: patient-specific; PF: pressure-gradient force; Repr.: representative; VF: virtual-mass force; WK: Windkessel; Y-90: Yttrium-90. *Simulation time depends on the workstation; for further information on the workstation refer to the cited reference.
